# ANXA1 inhibits miRNA-196a in a negative feedback loop through NF-kB and c-Myc to reduce breast cancer proliferation

**DOI:** 10.18632/oncotarget.8875

**Published:** 2016-04-20

**Authors:** Yi Yuan, Durkeshwari Anbalagan, Lay Hoon Lee, Ramar Perumal Samy, Muthu K. Shanmugam, Alan Prem Kumar, Gautam Sethi, Peter E. Lobie, Lina H.K. Lim

**Affiliations:** ^1^ Department of Physiology, Yong Loo Lin School of Medicine, National University Health System (NUHS), National University of Singapore (NUS), Singapore; ^2^ Department of Pharmacology, Yong Loo Lin School of Medicine, NUHS, National University of Singapore, Singapore; ^3^ Cancer Science Institute of Singapore, National University of Singapore, Singapore; ^4^ School of Biomedical Sciences, Curtin Health Innovation Research Institute, Curtin University, Perth WA, Australia; ^5^ National University Cancer Institute, NUHS, Singapore; ^6^ Department of Biological Sciences, University of North Texas, Denton, Texas, United States of America; ^7^ NUS Immunology Program, Life Sciences Institute, NUS, Singapore

**Keywords:** microRNAs, breast cancer, annexin 1, pri-miR-196a, c-myc, Immunology and Microbiology Section, Immune response, Immunity

## Abstract

MiRNAs are endogenous ~22 nt RNAs which play critical regulatory roles in a wide range of biological and pathological processes, which can act as oncogenes or tumor suppressor genes depending on their target genes. We have recently shown that ANXA1 inhibits the expression of miRNAs including miR196a. Here, we show that miR196a was highly expressed in ER+ MCF-7 breast cancer cells when compared to normal mammary gland cells, with expression levels negatively correlating to ANXA1. ANXA1 inhibits the biogenesis of oncogenic miR-196a by suppressing primary-miR196a indirectly through the stimulation of c-myc and NFkB expression and activity in breast cancer cells. In a negative feedback loop, miR-196a directly inhibits ANXA1 and enhances breast cancer cell proliferation in vitro. Finally, miR196a promotes breast tumor growth *in vivo*. This study reports a novel regulatory circuit between ANXA1, NF-kB, c-myc and miR-196a which regulates breast cancer cell proliferation and tumor growth.

## INTRODUCTION

Cancer is a serious healthcare problem with global incidences of >10 million and ~6 million deaths annually [1]. This incidence will double and rough estimates are reported to be 17 million deaths by 2030. Breast cancer is the second leading cause of cancer mortality in women and the incidence and mortality rate of breast cancer has been increasing worldwide [2]. In 2013, nearly 232,340 invasive breast cancer cases were diagnosed, and an estimated 64,640 new cases and 39,620 deaths were reported [3]. Currently available surgical and therapeutic interventions for breast cancer are limited [4]. In addition, all the existing drugs cause toxicity to some extent and results in serious adverse effects to patients [4,5]. Personalized medicine and new drugs under clinical trials are becoming practical limitations for drug development [6]. Nevertheless, the long-term prognosis of breast cancer treatment is not satisfactory. The elucidation of the molecular mechanisms underlying breast tumorigenesis and metastasis will be useful in the new discovery of novel biomarkers for the development of novel therapeutic interventions.

In the past decade, microRNAs (miRNAs) have emerged and play an important role in several aspects of tumor growth, development, metastasis, drug resistance and growth [7,8]. MiRNAs are a group of non-coding RNAs that have been shown to regulate several genes involved in various cellular processes including proliferation, differentiation and apoptosis [9]. miRNAs are approximately 22-nucleotide short non-coding RNAs which have been shown to be deregulated in cancer, including human breast cancer [9,10]. Recently, polymorphisms in miRNAs binding sites in target genes have been identified to increased risk of breast cancer in certain populations [11].

MiR-196a plays a significant role in oncogenic function in cancer such as inhibiting tumor suppressors and exhibited oncogenic effects. It has also been reported that miR-196a can directly target HOXA5 [12], HOXC8 [13] and Annexin-1 (ANXA1) [14]. Through the inhibition of HOXA5, miR-196a has been shown to promote cell invasion. However, the ectopic expression of miR-196a is associated with reduced cell migration, suggesting that the function of miR-196a in cancer may be tissue and cell type-specific.

ANXA1 was originally found to inhibit eicosanoid synthesis by inhibiting phospholipase A_2_ (PLA_2_) activity [15]. ANXA1 is implicated in many physiological processes, such as cellular transduction, inflammation, phagocytosis, proliferation, differentiation, and apoptosis [16]. We have previously reported that ANXA1 can increase NF-KB activity promoting breast cancer migration and metastasis [17] and ANXA1 can regulate miR expression and function in breast cancer [18]. Through ANXA1 targeting, miR-196a has been reported to promote cell growth in esophageal cancer cells [14], inhibit VEGF-mediated cell migration and angiogenesis in endothelial cells [19] and promote an oncogenic effect in head and neck cancer cells [20].

We have previously reported through microarray studies that ANXA1 can inhibit the expression of multiple miRNAs [18], and in this study we investigated the association between miR-196a and ANXA1 and their relevance to breast cancer cell proliferation and growth.

## RESULTS

### miR-196a expression in breast cancer cells

**Figure 1:**
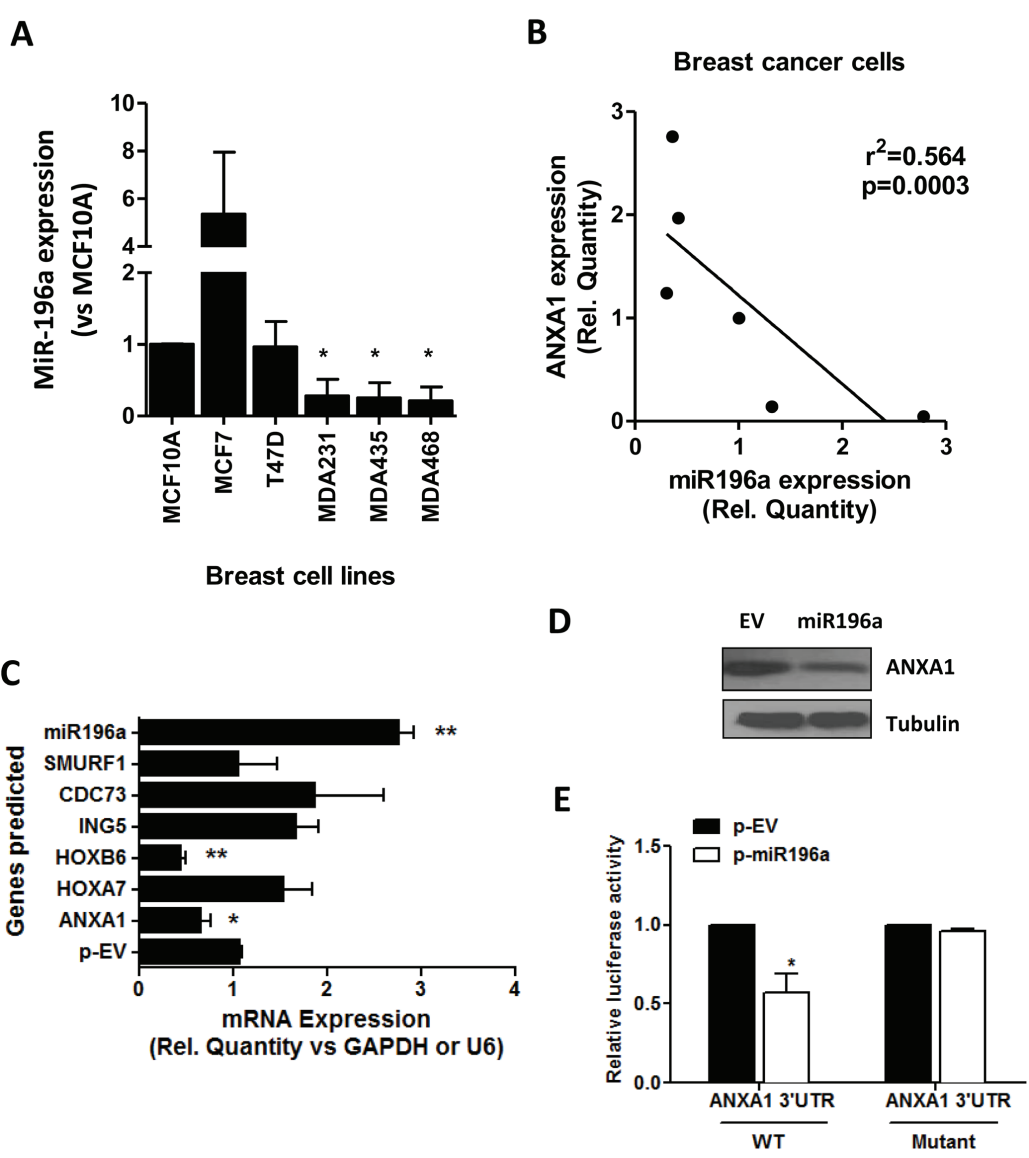
miR196a expression in breast cancer cells and targets ANXA1 **A.** miR196a expression was quantified by qPCR in 5 breast cancer cell lines and 1 breast epithelial cell line MCF10A. Results are represented as a fold change in mRNA levels after normalizing to U6 and comparing to MCF10A. **B.** ANXA1 expression and miR196a expression were plotted and a linear correlation was derived. **C.** Predicted targets of miR196a were quantified using qPCR and normalized against the housekeeping gene. **D.** ANXA1 protein expression was analyzed using western blot in MCF7 cells transfected with empty vector (EV) or miR196a plasmids. **E.** The luciferase reporter containing wild-type or mutant ANXA1 3′-UTR was co-transfected into HEK293T cells with miR-196 plasmids and control. Results shown are mean ± SEM of three independent experiments. *P < 0.05 **P < 0.01 *vs* the indicated controls.

The basal level of miR196a in a panel of breast cell lines including the breast epithelial cell line, MCF10A, the estrogen receptor positive MCF7 and T47D, and the triple negative basal-like breast cancer cell lines MDA-MB-231, MDA-MB-468 and MDA-MB-435 was first analyzed. Compared to MCF10A cells, expression levels of miR196a was high in MCF7 cells and lower in the basal like cell lines (MDA-MB-231, MDA-MB-468 and MDA-MB-435; Figure 1A). ANXA1 expression was previously reported to be low in MCF7 cells [17], therefore a significant negative correlation exists between ANXA1 and miR196a (Figure 1B).

We predicted that 6 genes including ANXA1 would be regulated by miR196a using 4 prediction softwares (TargetScan, MiRanda, Diana MicroT and PicTar), and confirmed the reduction of these genes (ANXA1, HOXA7, HOXB7, ING5, CDC73 and SMURF1) by miR-196a in breast cancer cells using qPCR analysis. Ectopic expression of miR196a reduced the expression of ANXA1 and HOXB6, while it did not change the expression of the other genes (Figure 1C). Similarly, ANXA1 protein expression was lower in MCF7 cells transfected with a plasmid overexpressing miR196a (Figure 1D). To prove that miR-196a directly targets ANXA1 in breast cancer cells, a wild-type 3′-UTR construct of ANXA1 and mutant 3′-UTR of ANXA1 construct was used with co-transfection of miR-196a vectors. MiR-196a decreased the activity of the luciferase reporter containing the wild-type 3′-UTR of ANXA1 mRNA. However, no significant difference in the activity of the luciferase reporter containing the mutant 3′-UTR of ANXA1 mRNA was observed (Figure 1E).

### ANXA1 inhibits miR-196a expression

**Figure 2:**
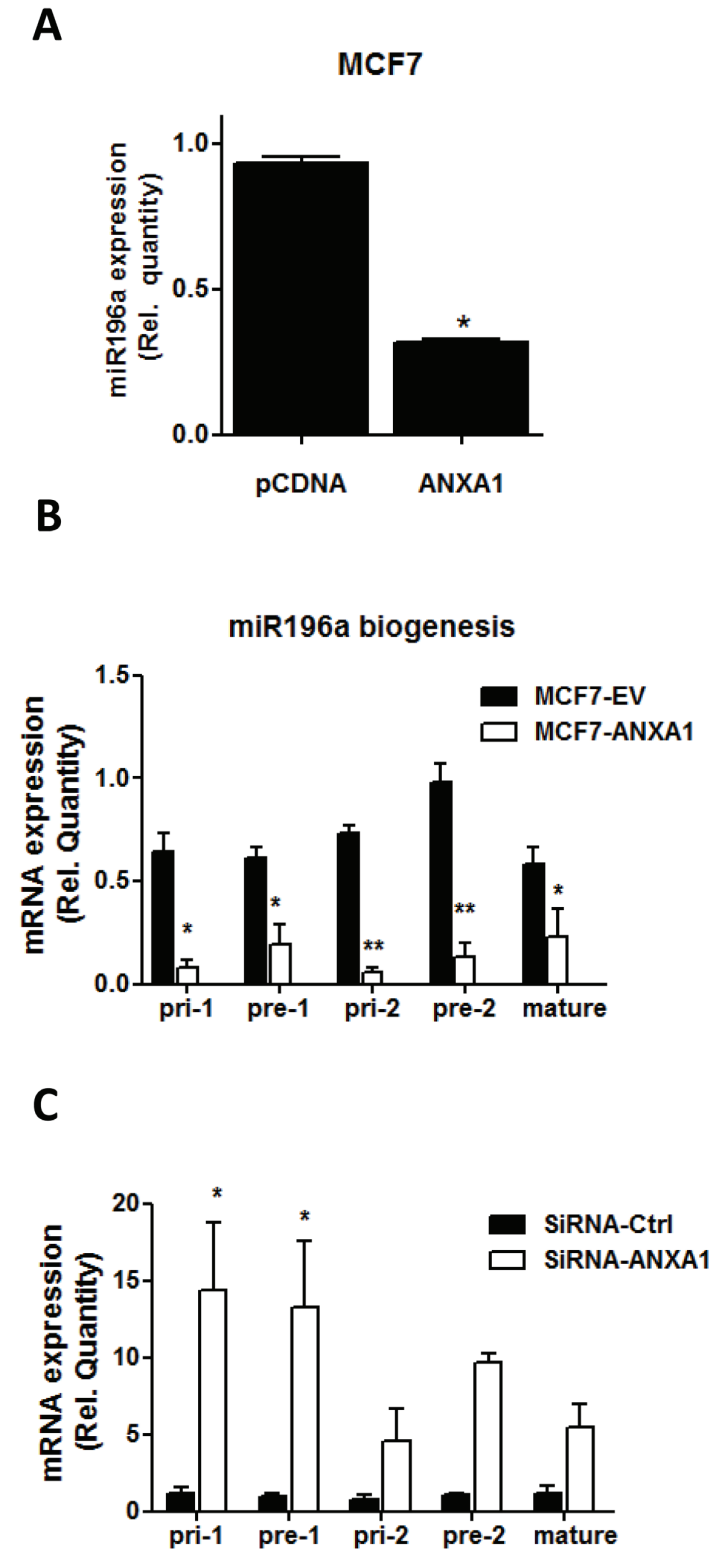
ANXA1 inhibits miR196a biogenesis **A.** MiR-196a expression in MCF7 cells and MCF7 cells overexpressing ANXA1 was quantified by qPCR. Results are represented as a fold change in mRNA levels after normalizing to U6. **B.** Primary miR-196a, precursor miR-196a and mature miR-196a expression levels were quantified by qPCR in MCF7 cells after overexpression or silencing of ANXA1. *P < 0.05. **P < 0.01 *vs* relevant controls.

We recently reported using microarray analysis that when ANXA1 was stably overexpressed in MCF7 cells, a number of microRNAs were decreased, including miR-196a which was down-regulated approximately 3 fold ([18], GEO GSE54439). These results were confirmed using qPCR which demonstrated that miR-196a expression is decreased in ANXA1 overexpressing MCF7 cells (Figure 2A). However, if and how ANXA1 regulates miR biogenesis is unclear. Therefore, the suppression of primary-miR196a (Pri), precursor-miR196a (pre) and mature miR-196a expression by ANXA1 was quantified using real-time qPCR in MCF7 cells overexpressing ANXA1. Overexpression of ANXA1 significantly reduces the expression of pri-miR196a-1 and pri-miR196a-2, pre-miR196a-1 and pre-miR196a-2 and mature miR-196a when analyzed with real time PCR (Figure 2B), while silencing ANXA1 with siRNA resulted in an increase in pri-miR196a-1 and pre-miR196a-1 (Figure 2C). This data indicates ANXA1 can inhibit miR biogenesis upstream of mature and precursor miR196a, thus reducing the transcription of miR-196a.

### C-Myc and p65 inhibit pri-miR196a transcription

**Figure 3:**
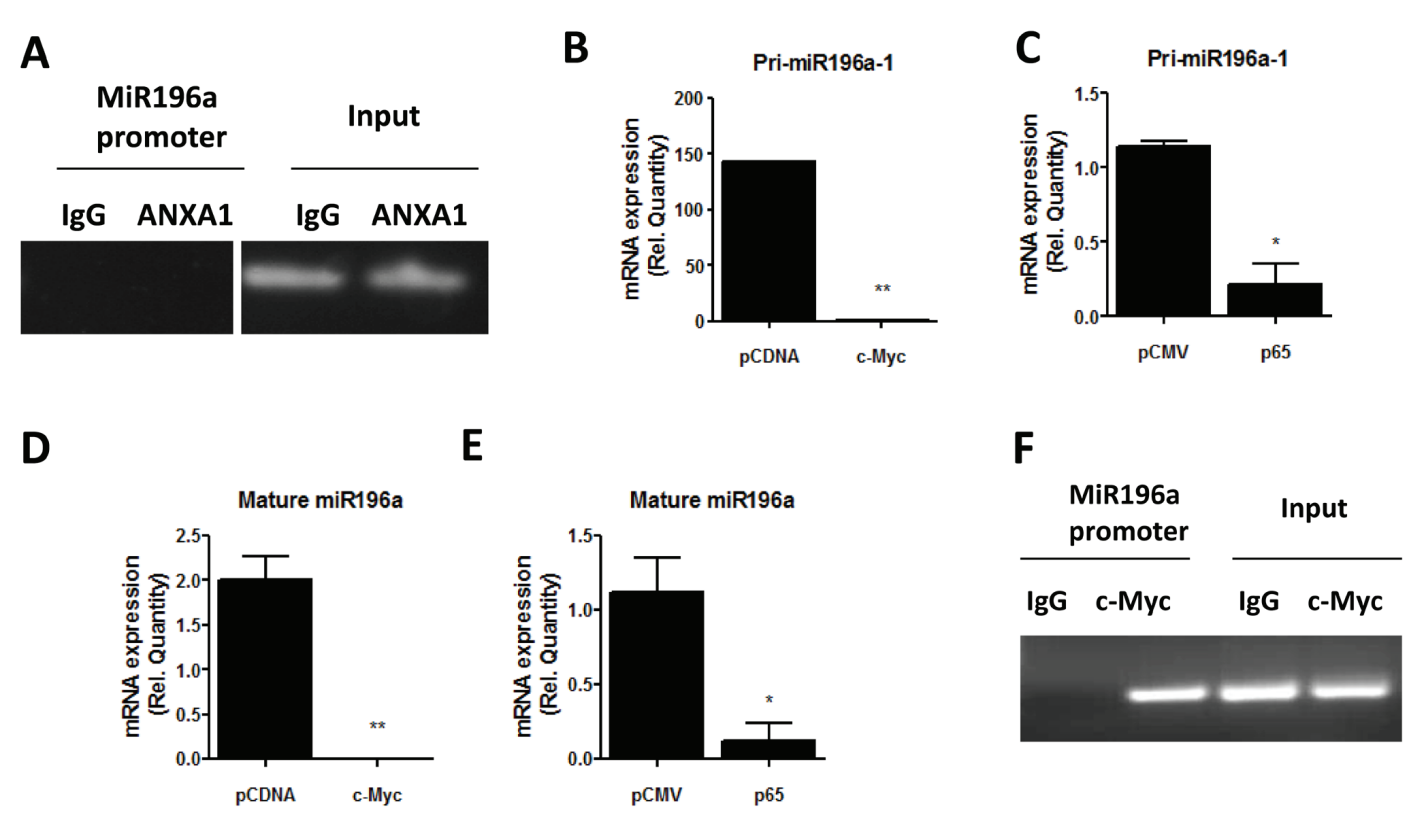
c-myc and NF-KB inhibit miR196a expression **A.** ANXA1 binding to the promoter of miR196a was analyzed using CHIP. **B.**-**E.**. MCF7 cells were transfected with c-myc or p65 or empty vector (EV) control plasmids, and the level of pri-miR-196a or mature miR196a was quantified by qPCR. *P < 0.05, **P < 0.01 *vs* empty vector control. **F.**. Binding of c-Myc to the promoter of miR-196a was analyzed using CHIP.

CHIP assay was performed to investigate whether ANXA1 binds to the promoter of miR-196a to inhibit transcription directly. ANXA1 did not bind to the promoter of miR-196a, suggesting that ANXA1 may inhibit the transcription of miR-196a indirectly (Figure 3A). Therefore, to assess if transcription factors such as c-myc or p65 NF-κB can inhibit the expression of pri-miR-196a, c-myc or p65 plasmids or their empty vector plasmids were expressed in MCF7 cells. Ectopic expression of c-myc and p65 significantly inhibits the expression of pri-miR196a-1 (Figure 3B, 3C). Furthermore, this translates to an inhibition in the mature form of miR196a when c-myc or p65 was overexpressed (Figure 3D, 3E). These results demonstrate that the transcription factors c-myc and p65 can inhibit the biogenesis and transcription of mature miR196a. CHIP was performed to investigate whether c-myc inhibits transcription of miR-196a expression by binding to its promoter. As shown in Figure 3F, c-myc, but not the IgG control can be shown to bind to the promoter of miR-196a, confirming that c-myc can regulate miR196a transcription.

### ANXA1 enhances c-Myc and NFκB activity

**Figure 4:**
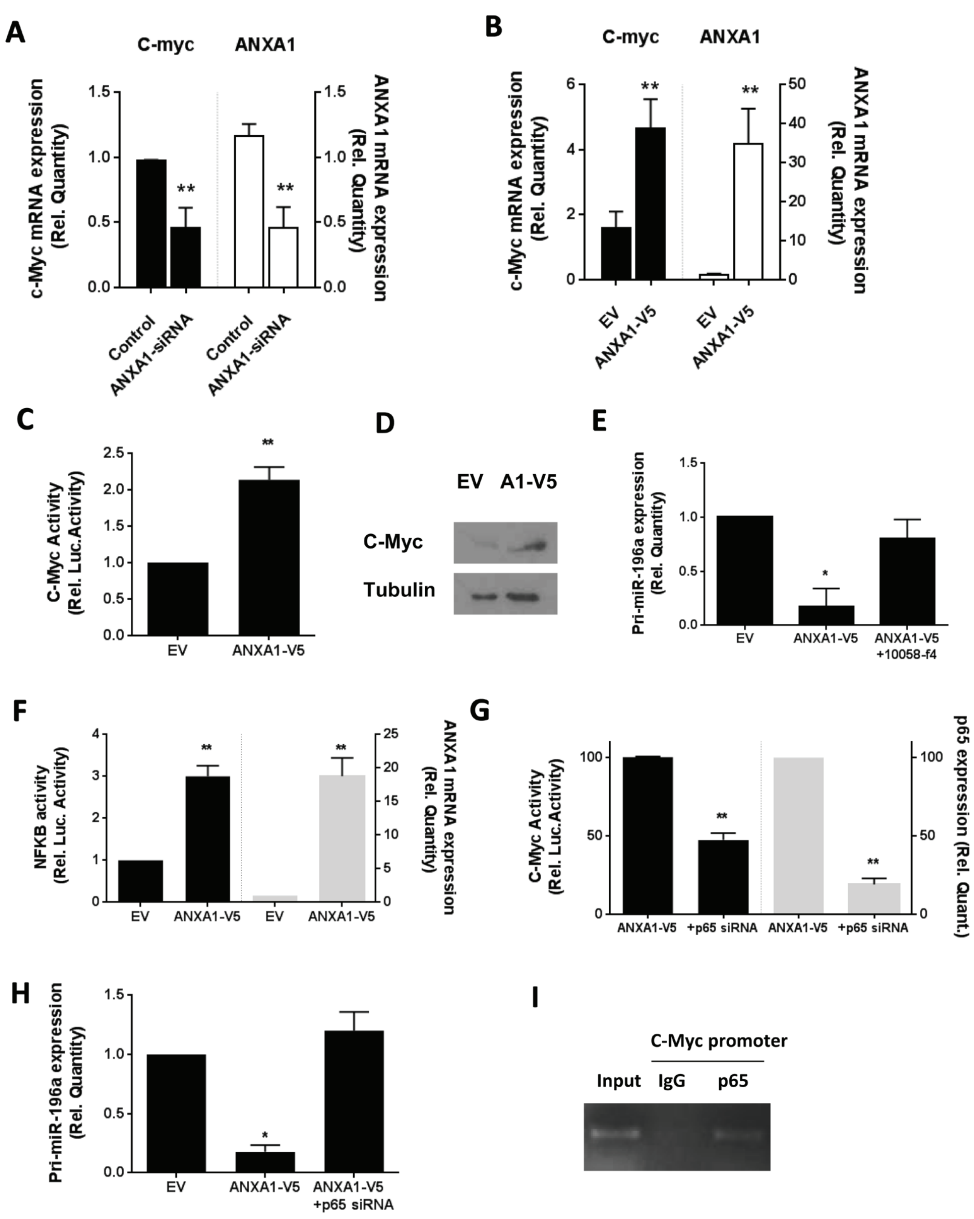
ANXA1 inhibits miR196a expression through c-myc and NF-KB **A.** MCF7 cells were transfected with control or ANXA1 siRNA and the levels of c-Myc or ANXA1 expression was determined using qPCR. **B.** MCF7 stably transfected EV or ANXA1-V5 cells were analyzed for levels of c-Myc or ANXA1 mRNA expression **C.** C-Myc promoter activity and **D.** c-Myc protein expression. **E.** MCF7 stably transfected EV or ANXA1-V5 cells were treated with the c-myc inhibitor 10058-f4 and the levels of primary miR196a was determined using qPCR. **F.** MCF7 stably transfected EV or ANXA1-V5 cells were analyzed for NFKB luciferase activity and ANXA1 expression. **G.** MCF7 stably transfected ANXA1-V5 cells were transfected with p65 siRNA and c-Myc activity and p65 expression, and **H.** primary miR196a expression was measured. *P < 0.05, **P < 0.01 *vs* relevant controls.

Overexpression of ANXA1 in MCF7 cells increased the *c-myc* mRNA expression, while silencing of ANXA1 resulted in lower *c-myc* gene expression, correlating with the level of ANXA1 present (Figure 4A, 4B). In addition, MCF7 cells overexpressing ANXA1 exhibited higher *c-Myc* gene expression (Figure 4C). C-myc protein level was also higher in MCF7 cells overexpressing ANXA1, as examined by western blot (Figure 4D). To determine if c-myc was involved in the suppression of miR-196a expression by ANXA1, ANXA1 stably transfected MCF7 cells were treated with c-myc inhibitor 10058-f4. Treatment of ANXA1-V5 MCF7 cells with 10058-f4 reversed the reduced expression of pri-miR196a-1 induced by ectopic expression of ANXA1 (Figure 4E).

### ANXA1 enhances c-myc activity via NFkB

MCF-7 cells overexpressing ANXA1 exhibited higher NF-κB luciferase activity, correlating with the higher expression of ANXA1 (Figure 4F). We next determined if NF-κB was involved in the modulation of miR196a transcription. MCF7 cells depleted of p65 indeed exhibited an inhibition in the ANXA1-induced reduction in pri-miR-196a expression (Figure 4G), indicating that both NFKB and C-Myc were playing a role in the modulation of pri-miR-196a expression.

To determine if NFkB could increase C-Myc activity, C-Myc activity was examined in MCF7 ANXA1-V5 cells silenced for p65. Interestingly, silencing p65 reduced C-Myc activity, correlating with the expression of p65 mRNA (Figure 4H). A ChIP assay confirmed that p65 could bind to the promoter of c-myc (Figure 4I), demonstrating a possible model where ANXA1 enhances activity of NFKB, which in turn, may increase the expression and activity of c-Myc, of which both inhibit the transcription of pri-miR196a.

### ANXA1 inhibits proliferation while MiR196a Promotes Proliferation and re-expression of ANXA1 reverses miR-196a proliferative function

**Figure 5:**
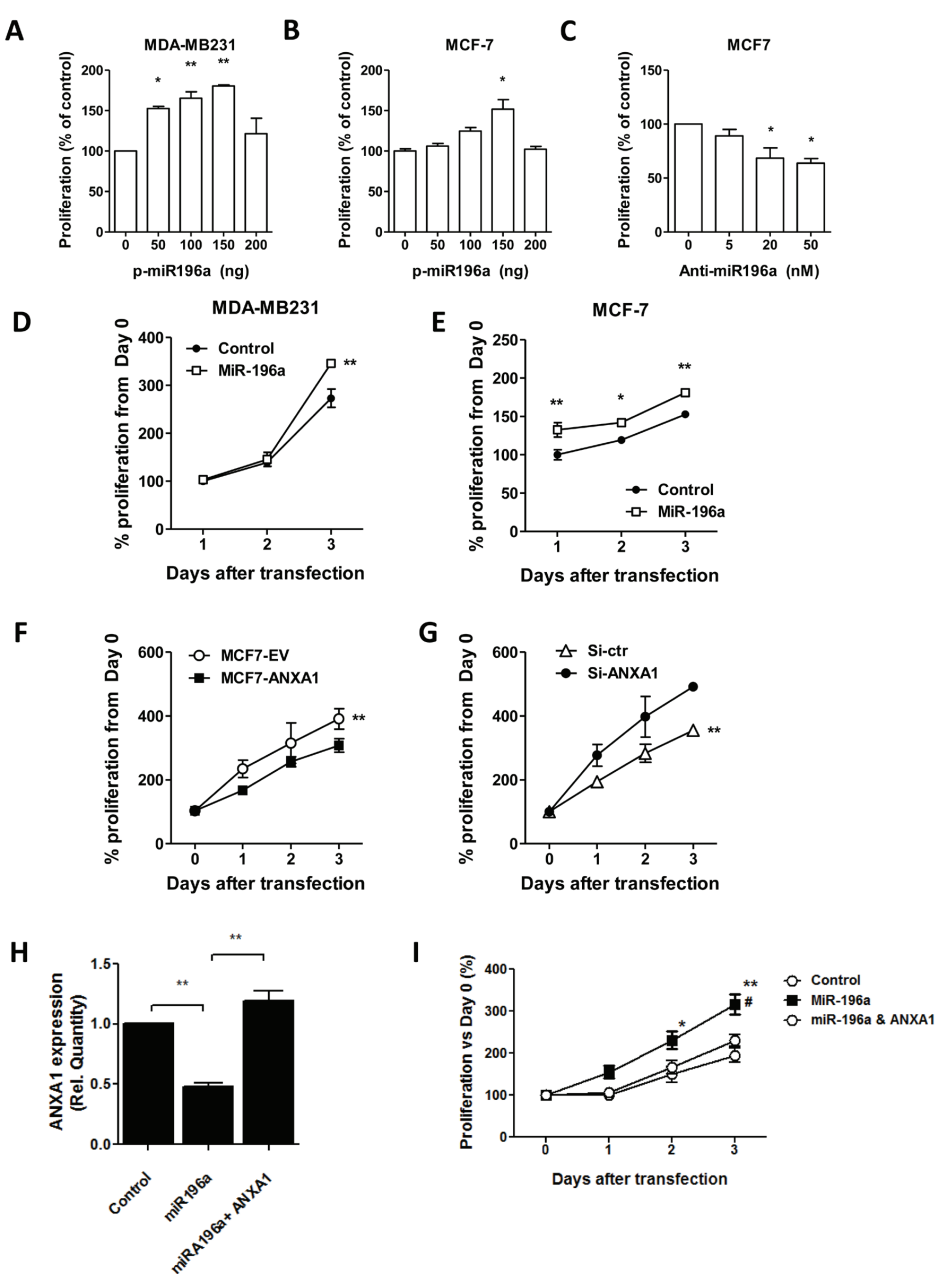
MiR-196a promotes breast cancer cell proliferation *in vitro* **A.**,**B.** miR-196a plasmids were transfected into MDA-MB-231 cells or MCF-7 cells at the indicated doses and proliferation was assessed. **C.** Anti-miR196a or negative control were transfected into MCF7 cells at the indicated doses and proliferation was assessed. **D.**,**E.** 100ng miR-196a plasmids were transfected into MDA-MB-231 cells or MCF-7 cells at the indicated time points and proliferation was assessed. **F.** Proliferation was measured in MCF7 stably transfected EV or ANXA1-V5 cells **G.** MCF7 cells were transfected with control or ANXA1 siRNA and proliferation was assessed. **H.** MCF7 stably transfected EV or ANXA1-V5 cells were transfected with plasmids to miR196a and ANXA1 expression **I.** and proliferation was measured at the indicated days after transfection. *P < 0.05, **P < 0.01 *vs* relevant controls. # *P* < 0.05 ANOVA *vs* miR196a.

MDA-MB-231 cells, which express low levels of miR196a (Figure 5A) and MCF-7 cells, which expressed higher levels of miR196a were transiently transfected with increasing concentrations of miR-196a plasmids. MiR196a expression significantly increased MDA-MB231 cell proliferation at concentrations of 50-150ng, while only enhancing cell growth in MCF7 cells at 150ng plasmid concentration, possibly due to the high basal level of miR196a found in MCF7 cells (Figure 5B). In contrast, MCF7 cells were transiently transfected with increasing concentrations of anti-miR-196a nucleotides. In these experiments, anti-miR196a nucleotides inhibited the growth of MCF-7 cells significantly at 20 and 50 nM (Figure 5C & 5D). MiR196a enhances proliferation in a time dependent manner in both MDA-MB231 cells and MCF-7 cells (Figure 5E).

Next we determined if ANXA1 exhibits an opposite function on proliferation (ie, ANXA1 inhibits proliferation). Cells transfected with ANXA1 proliferated significantly slower than cells transfected with empty vectors assessed (Figure 5F). In contrast, MCF7 cells silenced with siRNA to ANXA1 exhibited higher growth rates compared to control cells (Figure 5G). This data indicates that while the miR196a enhances proliferation, ANXA1 inhibits proliferation.

MCF7 cells overexpressing ANXA1 were transfected with miR-196a and growth rates were measured. Co-transfection with ANXA1 reversed the inhibition of ANXA1 expression by miR196a (Figure 5G), and re-introduction of ANXA1 partially rescued miR-196a-promoted proliferation in MCF7 cells (Figure 5H). This data shows that miR-196a may promote MCF7 cell proliferation via down-regulation of ANXA1 expression.

### miR-196a promotes breast tumor growth *in vivo*

**Figure 6:**
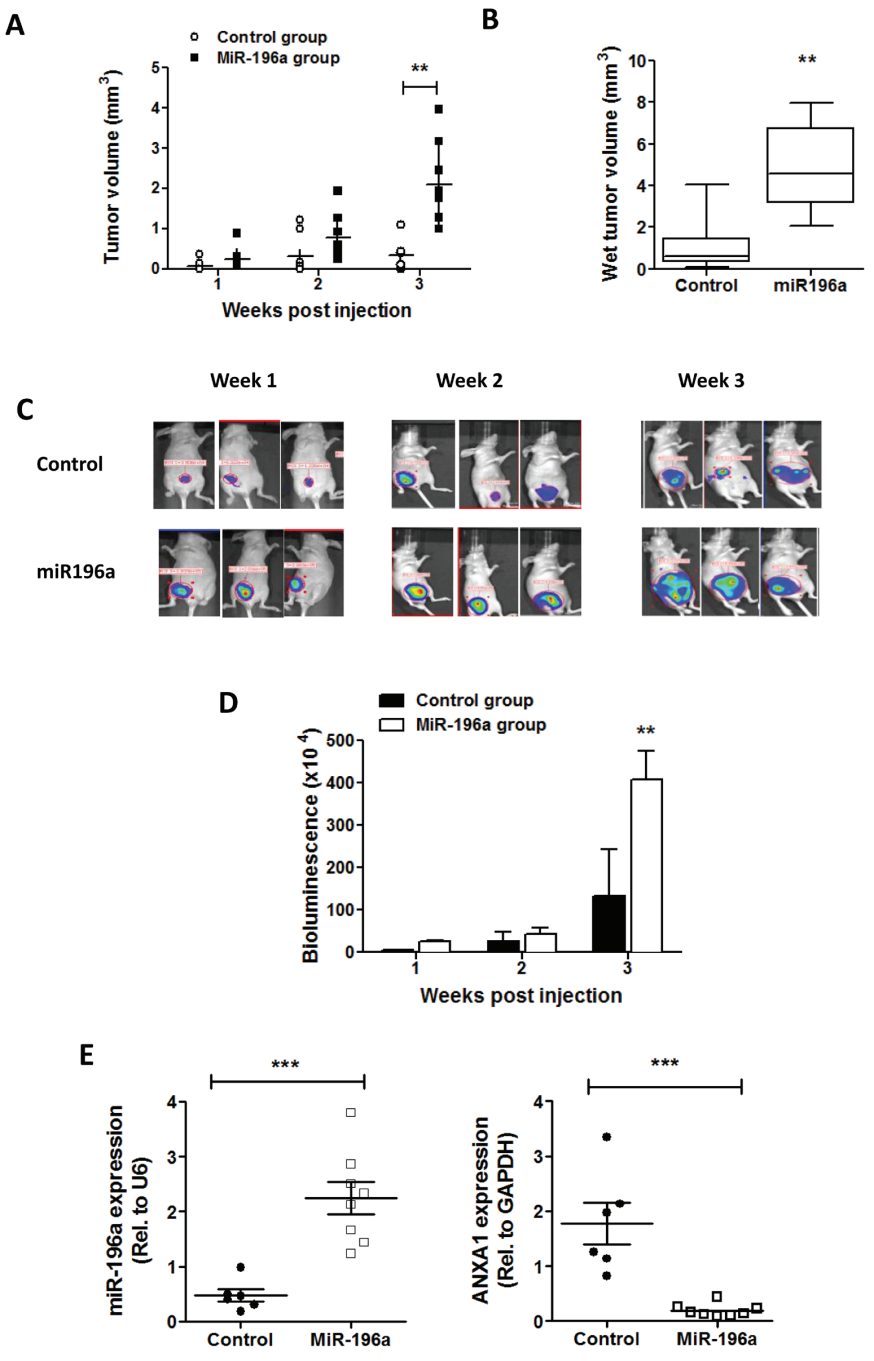
miR-196a promotes breast tumor growth *in vivo* BALB/c nu/nu athymic mice were injected with control MDA-MB231-luc cells or cells stably overexpressing miR196a into the mammary fat pad. **A.** Tumor size was measured every week post injection and tumor volume was calculated by LxWxW/2. **B.** Tumor volumes from tumors excised at week 3 were calculated by LxWxW/2. ** *P* < 0.01 *vs* control group. **C.** Bioluminescence imaging for mice from control group and miRNA group at week 1,2,3 post-injection. **D.** Quantification of bioluminescence data in tumor samples. **P < 0.01 *vs* control group. **E.**,**F.** The expression of miR-196a or ANXA1 was conducted by qPCR in tumors from control and miRNA groups. Results are represented as a fold change in mRNA levels in tumors normalized to U6 or GAPDH, respectively. *** *P* < 0.001 *vs* control group.

Finally, to verify the oncogenic microRNA effect of miR-196a in vivo, MDA-MB-231-luc breast cancer cells which harbor a luciferase promoter was stably transfected with miR-196a plasmids (or) empty vectors. These cells were injected into the mammary gland of female athymic nude (Nu/Nu) mice. After the injection, tumor growth was measured by manual measuring and bioluminescence imaging every week (1, 2 and 3 weeks). Tumor growth was significantly higher in miRNA group compared to control group injected with empty vector plasmids (Figure 6A). Similarly, the bioluminescence imaging also confirmed that the group injected with miR-196a- MDA-MB-231-luc exhibited increased luminescence (Figure 6B). Figure 6C shows that tumors collected from control mice at week 3 post-xenograft were smaller than tumors collected from the miR196a group. The expression of miR-196a in tumors was confirmed in the 2 groups, showing that miR196a was indeed higher in the tumors from the miR196a group. (Figure 6D). This indicates that the miR-196a directly targets ANXA1 in breast cancer cells. Finally, we analyzed the expression of miR196a and ANXA1 in the tumors, and it can be seen that tumors from the miR196a group express low levels of ANXA1 while expressing high levels of miR196a (Figure 6E).

## SUMMARY

MicroRNA-196a forms a negative regulatory circuit with Annexin-A1 where ANXA1 inhibits miR196a biogenesis through NF-KB and c-myc activation. In turn, miR196a inhibits ANXA1 transcription directly through its 3′UTR. miR196a may play a role in the anti-proliferative effects of ANXA1.

## DISCUSSION

Our present study is focused on elucidating the mechanism of suppression of miRs by ANXA1. We have previously reported that ANXA1 can inhibit the expression of a number of miRNAs including miR196a. Deregulation of miRNAs is emerging as a major aspect of cancer etiology because of their capacity to direct the translation and stability of targeted transcripts that can dramatically influence cellular physiology [21].

It was previously reported that miR-196a is up-regulated in T47D and down-regulated in MDA-MB-231 and MDA-MB-435 cells [14], which is in line with our findings in MDA-MB-231 and MDA-MB-468 triple negative cells. Global microRNA profiling studies showed the miR-196 is increased in formalin-fixed paraffin-embedded breast cancer tissues [22]. Additionally, the common sequence variants of rs11614913 CC genotype in miR-196a-2 increases breast cancer risk which provides useful information for the early diagnosis and prevention of breast cancer [23]. Aside from miR196a, several miRNA polymorphisms have been reported to suppress or activate miRNAs processing or miRNAs-mRNA interactions [24] and single nucleotide polymorphisms (SNPs) located in miRNAs was associated with increased risk of breast cancer susceptibility [25]. Moreover, SNPs studied with special reference to miR196a (rs3746444, rs11614913, rs1044129) can be used as genetic markers to predict not only breast cancer susceptibility but also prognosis for early detection [26].

Several studies have reported the oncogenic function of miR-196a in various cancer cells [12,27]. Our results also confirm that miR-196a plays an oncogenic role in breast cancer cells as over-expression of miR-196a promotes cell proliferation whereas the inhibiting miR-196a using anti-miR suppresses breast cancer cell proliferation. Furthermore, our *in vivo* experiments demonstrate that forced expression of miR-196a in MDA-MB-231 cells induced significantly higher tumor growth confirming that miR-196a promotes breast cancer growth. The oncogenic role of miR-196a and the tumor-suppressive role of anti-miR-196a in breast cancer cells may be of therapeutic potential in breast cancers.

In the present study, we show a negative regulatory circuit between ANXA1 and miR196a where ANXA1 is a target of miR196a and can also inhibit primary miR196a expression. The inhibition of ANXA1 expression by miR196a is not novel as other groups have described that ANXA1 may be lost in cancer due to inhibition by miR196a [14]. We have previously reported that ANXA1 reduced miRNA expression in breast cancer cells [18], but the mechanism was unknown. We show here that pri-miR-196a, pre-miR-196a and mature miR-196a were all inhibited by ANXA1. These findings suggest that ANXA1 inhibits the miRNA biogenesis pathway via the transcription of miR-196a upstream of the enzymes Drosha, Pasha and exportin. ANXA1 does not bind directly to the promoter of miR-196a directly but may act via an indirect mechanism, through transcription factors, namely c-myc and NF-κB. Activation of NF-κB by ANXA1 was previously shown by us to result in the constitutive activation of NF-κB and subsequent effects on migration and metastasis of breast cancer cells [17]. In addition, we have also shown that ANXA1 can enhance ERK activity and RhoA activity in breast cancer [28].

C-myc is an oncogenic transcription factor that regulates a wide range of cellular processes and the association between microRNAs and c-myc well known [29]. We identified that c-myc and NF-κB reduced pri-miR-196a expression. C-Myc may inhibit microRNA expression via several mechanisms. Firstly, c-myc can bind to the promoter of miRNA genes and inhibit the transcription of pri-miRNAs transcripts. Secondly, c-myc inhibits miRNA processing by transcriptional repression of Drosha and has been shown to decrease let-7 processing by transcriptional regulation of Lin 28 [30]. It is interesting and surprising that c-Myc, which is an oncogenic transcription factor inhibits the expression of a oncogenic miR. Obviously, miR196a is not the only miR which can be inhibited by c-Myc and c-Myc can also be regulated through epigenetic mechanisms [31]. NF-κB has been reported to enhance the expression of other microRNAs such as miR-146a [32] and miR-34a [33]. Several studies have been reported the involvement and elevated levels of NF-κB DNA-binding activities in several cancers, inflammatory and autoimmune diseases [34].

Importantly, our study is the first to report that ANXA1 and miR-196a form a regulatory loop to modulate breast cancer cell proliferation. Our results corroborate with the double negative loop of miRNA and target genes which have been reported in other cancers by several groups. MiR-422a inhibits hepatocellular carcinoma (HCC) growth and metastasis through the inhibition of FOXG1/Q1/E1, which in turn inhibits the expression of miR-422a, forming a double negative feedback loop [35]. In a similar study, c-Myc increases miR-17-5p expression to inhibit metastasis of HCC, while identified as a target of miR-17-5p [36]. Moreover, NF-κB can inhibit miR146a, which in turn can inhibit the expression of NF-κB through TRAF-6 [37]. These microRNA-mediated feedback loops are believed to behave as bi-stable switches to regulate downstream processes [38].

In summary, we show that ANXA1 is a target of miR196a and in turn can also inhibit miR196a transcription, inducing a negative feedback loop to promote breast cancer growth and proliferation. Previous microarray data [18] and current data demonstrate that ANXA1 inhibits miR-196a expression, while co-expression of ANXA1 and miR-196a reverses both the miR-196a-induced cell proliferation and ANXA1-suppressed cell proliferation. We show that this is through the activation of NF-κB and c-Myc. All these data suggest that inhibiting onco-miR-196a expression may be one of the possible mechanisms by which ANXA1 suppresses cell growth in breast cancer cells, and on the other hand, miR-196a may induce breast cancer cell proliferation by targeting ANXA1.

## MATERIALS AND METHODS

### Cell culture

Human breast cell lines MCF10A, MCF7, T47D, MDA-MB-231, MDA-MB-435, MDA-MB-468 were obtained from American Type Culture Collection (ATCC, Manassas, VA, USA). Human breast cancer cell lines MCF7, MDA-MB-231, MDA-MB-435, MDA-MB-468 were grown as monolayers in Dulbecco's Modified Eagle's Medium (DMEM, Serana, Australia), while T47D was cultured in Roswell Park Memorial Institute (RPMI) medium supplemented with 10% heat-inactivated fetal bovine serum (FBS, Biowest LLC, Kansas, MO, USA), 1% penicillin-streptomycin (GE Healthcare Life Sciences, HyClone Laboratories, Utah, USA)at 37°C in a humid atmosphere containing 5% CO_2_. Human mammary gland epithelial cell line MCF10A was grown in DMEM/F12 with 15 mM HEPES buffer (4-(2-hydroxyethyl)-1-piperazineethanesulfonic acid), 5% horse serum, 10 μg/ml insulin, 20 ng/ml epidermal growth factor (EGF), 0.5 μg/ml hydrocortisone and 1% P/S. The MDA-MB-231-luc cell is a metastatic human breast cancer luciferase cell line cultured in DMEM with 20% FBS. All cells were grown in a humid atmosphere containing 5% CO_2_ at 37°C (Thermo Fisher Scientific Inc, MA, USA), The cell lines were regularly authenticated through cell morphology monitoring, growth curve analysis and species verification.

### Transfection

MicroRNA (miR-196a) sequences were cloned into pSilencer 4.1 CMV neo, while the psiCheck-3′UTR-ANXA1-wt and pSiCheck-3′UTR-ANXA1-mut were a kind gift from Dr Huot from Le Centre de recherche en cancérologie de l'Université Laval and Centre de recherche du CHUQ, Québec, Canada. The miR-196a inhibitor was purchased from Qiagen (Hilden, Germany). Transfection of the plasmids was performed by using Turbofect transfection reagent (Fermentas Inc, Hanover, USA) according to the manufacturer's instructions. Cells were seeded and incubated at 37°C (5% CO_2_) for overnight, until the cells are 80-90% confluent, plasmids and transfection reagent were mixed in serum and antibiotics free medium for 24h.

### SiRNA and anti-miR transfection

siRNA controls and siRNA-ANXA1 was obtained from Santa Cruz Biotechnology, Inc., California, USA was used according to the manufacturer's protocol. The siRNA and transfection reagent were mixed with serum as well as antibiotics free medium. After 30 min of incubation, the mixture was added to pre-washed cells and incubates around 5 hrs before changing to normal growth medium, siRNA oligo-scrambled (Santa Cruz Biotechnology, CA, USA) used as control at the similar concentrations. After transfection at 36-48 hrs, the cells were harvested for western blotting (or) qPCR analyses (or) other experiments.

### Extraction of RNA and qRT-PCR

Total RNA was extracted from the cells by using TRIzol reagent (Invitrogen, Carlsbad, CA, USA) according to the manufacturer's protocol. The miRNA was also extracted by using NucleoSpin miRNA kit (Macherey-Nagel, Postfach, Duren, Germany) according to the manufacturer's protocol. The quantitative and qualitative RNA analyses were performed by using NanoDrop 1000 spectrophotometer (Thermo Fisher Scientific, Massachusetts, USA). Total RNA (1ug) was used to synthesize cDNA by using a reverse transcription kit (GoTaq^®^ qpCR master mix, Promega Corporation, Madison, USA). MicroRNA (500 ng) was used to synthesize cDNA and Real-time polymerase chain reaction (RT-PCR) with miRCURY LNA^TM^ system performed according to the manufacturer's protocol. The results were normalized to the expression of glyceraldehyde-3-phosphate dehydrogenase (GAPDH) or U6 spliceosomal RNA.

### Western blot analysis

Cells were washed twice with ice cold 1 x phosphate buffer saline (PBS). Proteins were extracted from the cells by using RIPA lysis and extraction buffer containing 50 mM sodium chloride (NaCl), 0.1% NP-40, 0.5% sodium deoxycholate, 0.1% SDS and 50 mM Tris-HCl) supplemented with protease inhibitors cocktail (Roche Inc, Nutley, NJ, USA) and sodium orthovanadate (Sigma Co, St Louis MO, USA). Protein concentration was estimated according to the Bradford's protein assay (1x) system (BioRad Laboratories, Hercules, California, USA). Proteins (50 μg) were loaded onto 10% denatured polyacrylamide gel and separated by SDS-PAGE. Separated proteins were transferred to nitrocellulose membrane (BioRad, CA, USA). The membranes were blocked with 5% non-fat dry milk in 1 x Tris-buffered saline and Tween 20 (TBST) of 0.1% v/v and incubated with Annexin-A1, c-Myc, p65 primary antibody (Cell Signaling Technology Inc, Danvers, MA, USA) overnight at 4°C and monoclonal alpha-tubulin (Sigma-Aldrich Co, St. Louis, MO, USA) and room-temperature with secondary antibody. The immunoreactive protein bands were detected by chemiluminescent reagent (GE Healthcare BioAmersham, Pittsburgh, USA). The blots were exposed and developed with Konica Minolta SRX-101A tabletop processor.

### Cell proliferation assays

5000 cells transfected with miR-196a or anti-miR-196a or negative controls cells were harvested and plated in 96-well plates containing normal media until visibly confluent (80%). The cell proliferation as well as cell counting assays was determined using CellTiter 96^®^ (aqueous one solution cell proliferation) assay kit according to the manufacturer's instruction (Promega). Plates were incubated at 37°C for 2hr before reading OD at 490nm.

### Dual luciferase reporter assay

Cells were transfected when they reached 80% confluency. The cells were transfected with 100 ng of wild type or mutant plasmids in serum-free DMEM medium with and without miRs. Cells were incubated for 24 hours. Luciferase activity was determined using Dual- Luciferase^®^ Reporter Assay System (Promega). Cells were lysed in 120 μl of 1 x lysis buffer, centrifuged at 10000 g for 10 min and supernatants were collected. The results were expressed as relative promoter luciferase activity compared to controls after normalising for Renilla activity and protein concentration. Luminescence was measured using a spectrophotometer (Perkin Elmer VICTOR3™ V Multilabel Counter Model 1420).

### Chromatin immunoprecipitation

Human breast cancer (MCF7) cells (cell density 3 × 10^6^) were seeded in 10-cm plates and transfected with c-myc plasmids. The cells were fixed with 1% formaldehyde at room temperature after 48 hrs post transfection and neutralized with glycine. The cells were collected, resuspended in CHIP lysis buffer and sonicated (Vibra-Cell™, Newtown, USA). Samples were incubated with protein-G beads that had been pre-incubated with 4-10 μg of anti-cmyc antibody (Gene tex Inc, San Antonio, Texas, USA) or negative control IgG (Sigma-Aldrich Co, St. Louis, MO, USA). Immunoprecipitates were washed by using washing buffer and reverse-cross-linked. Then the DNA was purified by using PCR purification kit purchased from MACHEREY-NAGEL, Duren, Germany. Finally, the purity of the DNA was quantified by using NanoDrop, performed PCR and data analyzed.

### *In vivo* experiments

6-8 weeks old BALB/C nude female mice were obtained from InVivos (Singapore). The nude mice were maintained under specific-pathogen-free conditions in the Animal holding Units, Centre for Life Sciences, National University of Singapore (NUS), Singapore. This study was performed in strict accordance with the recommendations and experimental protocol approved by the Institute Animal Care and Use Committee (IACUC Protocol No: R13-5608) at Yong Loo Lin School of Medicine, NUS. Sixteen BALB/C nude mice were randomized into two groups (8 mice per group, n = 8). The first group of nude mice was subcutaneously injected by stably transfected MDA-MB-231 luciferase cells (8 ×10^6^) with negative vector into the mammary fat pad. The second group of mice was sc. injected by MDA-MB-231 luciferase cells (8 ×10^6^) stably transfected with miR-196a. The size of the tumors was measured every week (1, 2 and 3 weeks) by a bioluminescence imaging assay using the Xenogen IVIS Spectrum Iamging System (Caliper Life Sciences) and manual measurement by Vernier caliper. All the mice were sacrificed after the tumor reached 100 mm^3^ size and collected lung as well as breast tumors collected sacrificed for various biochemical assays.

### Statistical analysis

Results are the means ± SEM of three independent experiments performed in triplicate. Statistical comparisons between groups were made by using one-way ANOVA and unpaired two-tailed Student's t-test used for comparing two variables. The differences were considered statistically significant at *p < 0.05.
